# Modulation of the strength and character of HIV-specific CD8^+^ T cell responses with heteroclitic peptides

**DOI:** 10.1186/s12981-017-0170-y

**Published:** 2017-09-12

**Authors:** Kayla A. Holder, Michael D. Grant

**Affiliations:** 1 Division of BioMedical Sciences, Immunology and Infectious Diseases Program, Faculty of Medicine, Memorial University of Newfoundland, St. John’s, NL A1B 3V6 Canada; 2 Division of BioMedical Sciences, Faculty of Medicine, Memorial University of Newfoundland, 300 Prince Phillip Drive, St. John’s, NL A1B 3V6 Canada

**Keywords:** CD8^+^ T cell, HIV, Heteroclitic, Peptide, T cell receptor, MHC, Proliferation, IFN-γ, IL-2, Therapeutic vaccine

## Abstract

Chronic infection with human immunodeficiency virus (HIV) causes HIV-specific CD8^+^ T cell dysfunction and exhaustion. The strong association between non-progression and maintenance of HIV-specific CD8^+^ T cell cytokine production and proliferative capacities suggests that invigorating CD8^+^ T cell immune responses would reduce viremia and slow disease progression. A series of studies have demonstrated that sequence variants of native immunogenic peptides can generate more robust CD8^+^ T cell responses and that stimulation with these ‘heteroclitic’ peptides can steer responses away from the phenotypic and functional attributes of exhaustion acquired during chronic HIV infection. Incorporation of heteroclitic peptide stimulation within therapeutic vaccines could favour induction of more effective cellular antiviral responses, and in combination with ‘shock and kill’ strategies, contribute towards HIV cure.

## Background

Cytotoxic CD8^+^ T cells kill damaged, malignant or virus-infected cells through granule-relayed or receptor-mediated mechanisms. Recognition by, and activation of CD8^+^ T cells is governed via interaction between the T cell receptor (TCR) and antigen-derived peptides bound within cognate class I major histocompatibility complexes (MHC-I). Stabilizing derivations that fasten antigenic peptides to MHC (pMHC) molecules more securely, or enhance TCR binding to the pMHC complex, increase peptide immunogenicity [1, 2].

During early infection, human immunodeficiency virus (HIV)-specific CD8^+^ T cells are critical for limiting HIV replication in vivo [3]. Long term non-progressors maintain HIV-specific CD8^+^ T cells with a superior functional profile than those from progressors [4]. Thus, initially robust CD8^+^ T cell cytolysis and cytokine production wanes during progressive chronic HIV infection as T cells fail to recognize viral escape variants, become progressively exhausted and ultimately sustain dysfunctional immune responses against HIV [5, 6]. Maintaining HIV-specific CD8^+^ T cell potency would promote clearance of virus-infected cells, reduce viremia, and slow disease progression. Here, we review recent research that exploits the TCR:pMHC interaction axis to modulate HIV-specific CD8^+^ T cell responses in vitro and discuss potential therapeutic application of this approach.

## Variant peptides

Methods of augmenting TCR:pMHC complex avidity are varied and can involve either modifying the TCR or MHC-I complexes themselves, or creating peptide sequence variants [1, 2]. Augmenting T cell responses and effector functions via the latter method offers a less invasive and simpler approach to CD8^+^ T cell-based HIV vaccine therapy. As individual T cell clones recognize series’ of related peptides, constructing heteroclitic sequence variants that increase TCR:pMHC avidity, relative to reference peptides, can produce more potent CD8^+^ T cell responses [1]. Solinger et al. demonstrated the phenomenon of heteroclitic peptides as murine T cells primed with pigeon cytochrome *c* proliferated to a greater extent following restimulation with moth cytochrome *c* than with pigeon cytochrome *c* [7]. Mapping the T cell response to the immunodominant portion of the antigen affirmed that one family of clones can react equally well to either the priming peptide itself or to variant peptides encompassing amino acid (aa) substitutions, and that conservative modifications can support enhanced binding to MHC proteins or strengthen TCR:pMHC interactions [7].

Anchor modified heteroclitic peptides in the context of many MHC-I molecules have aa substitutions involving N- and C-terminus anchors that penetrate the floor of the MHC-I peptide-binding groove to secure the peptide position and orientation [2]. Anchor residue-modified peptides could bind within the MHC-I binding groove more strongly to enhance pMHC-I complex stability [8]. This higher avidity pMHC interaction subsequently requires less antigen to generate a CD8^+^ T cell response. As the TCR does not directly interact with buried anchor residues, substitutions here are unlikely to negatively affect TCR recognition, unless they have indirect effects on orientation of the peptide towards the TCR. For example, some modifications in anchor residues alter peptide conformation by creating a ‘bubble’, which can adversely affect the TCR recognition platform [9].

Heteroclitic peptides can also arise from conservative and semi-conservative aa substitutions at positions directly interacting with the TCR. Modifying the TCR recognition sequence appropriately should increase T cell sensitivity to antigen and substitution of aa at non-anchor positions was found to produce variant peptides more immunogenic than their native counterparts [10]. T cells responding to low concentrations of these variant peptides are high-avidity T cells, reflecting greater TCR:pMHC avidity invoked by appropriate modification of the TCR peptide contact interface [11, 12].

## HIV-specific CD8^+^ T cell response to variant peptides

Cytotoxic CD8^+^ T cells (CTL) are critical for controlling certain cancers, HIV and many other viral infections [12]. They predominantly eliminate infected cells through production and targeted release of cytotoxins [13]. Studies of HIV slow or non-progressors demonstrated strong cellular immune responses against HIV in association with class I human histocompatibility-linked leukocyte antigen (HLA) B*57 alleles [14]. Long-term non-progressors mount especially robust CD8^+^ T cell responses against HLA B*57-restricted HIV Gag protein peptide epitopes [14]. As CTL activity is maintained in chronic HIV infection with selective peptide stimulation in this genetically restricted context, identifying variant peptides that can efficiently induce and maintain CTL activity during progressive HIV infection across a broader genetic background could be an effective therapeutic vaccine strategy. In this context, several anchor-modified variants of the HIV reverse transcriptase (RT) peptide (ILKEPVHGV) enabled greater HIV-specific CD8^+^ T cell cytotoxic responses than stimulation with reference peptide [15, 16].

Cytokine production is instrumental to T cell development, differentiation and effector functions [17]. During chronic HIV infection, profound abnormalities in pro-inflammatory interleukin (IL)-2, tumour necrosis factor (TNF)-α, and interferon (IFN)-γ production arise sequentially as HIV-specific T cells approach exhaustion [5, 6]. T cell dysfunction in HIV infection represents a continuum spanning different degrees of dysfunctional cytokine production and reduced proliferation culminating in exhaustion [5, 6]. Variant peptide constructs can enhance ex vivo T cell cytokine responses of HIV-specific CD8^+^ T cells. Heteroclitic peptides with aa substitutions in the TCR recognition motif of immunodominant HIV Gag and Nef epitopes enhanced CD8^+^ T cell IFN-γ and/or IL-2 production in vitro, relative to the native peptides [18]. In some cases, TCR engagement with heteroclitic peptides affected IFN-γ or IL-2 production selectively, illustrating potential for heteroclitic peptides to exert qualitative effects on T cell cytokine production [18].

Memory CD8^+^ T cells producing IL-2 retain high proliferative capacity [4]. Any bias introduced towards CD8^+^ T cell IL-2 production after in vitro heteroclitic peptide stimulation could suggest that heteroclitic variant peptides could not only reconstitute antigen-specific T cell cytokine production and cytotoxicity, but also support clonotypic proliferation of HIV-specific CD8^+^ T cells no longer, or never activated by native peptides. Adegoke et al. examined HIV-specific CD8^+^ T cell proliferation in vitro using HIV-specific heteroclitic peptides previously shown to increase T cell cytokine responses. They observed increased HIV-specific CD8^+^ T cell proliferation against heteroclitic peptides in more than 25% of the cases where cytokine production was enhanced [19]. CD8^+^ T cell proliferation driven by either heteroclitic anti-tumour or self-peptides has also been observed, further demonstrating that apparent T cell insufficiency and even T cell tolerance can be overcome by using sequence variants of the reference peptides lacking desired activity [20–22].

As HIV-specific heteroclitic peptides can restore CD8^+^ T cell cytokine production and proliferation, probing their potential role in managing HIV-specific T cell exhaustion could also be informative. T cell exhaustion occurs with antigen persistence during malignancy or chronic infections and is defined by successive loss of IL-2, TNF-α and IFN-γ production, poor cytotoxic and proliferative capacity, and increased expression of inhibitory receptors such as programmed cell death (PD-1), cytotoxic T-lymphocyte-associated protein 4 (CTLA-4) or T cell immunoglobulin and mucin-domain containing-3 (TIM-3) [23–25]. Effective cancer immune therapies currently employ immune checkpoint-inhibitors targeting the inhibitory receptors associated with T cell exhaustion. Thus, application of strategies to renew exhausted HIV-specific CD8^+^ T cells could be a beneficial adjunct to current HIV treatment. In some cases, the same heteroclitic peptides that boosted HIV-specific CD8^+^ T cell proliferation also stimulated a proliferating population of HIV-specific T cells expressing less PD-1 than those proliferating following stimulation with native peptides [19]. With this impact analogous to that of checkpoint inhibitors, antigen-specific heteroclitic peptides could direct HIV-specific CD8^+^ T cell responses away from the exhaustive phenotype, potentially restoring clonogenicity, cytotoxicity and cytokine production to levels associated with long-term non progression.

## Implications in therapeutic vaccine

Synthetic peptide variants can enhance MHC interaction with the TCR by reducing adverse interactions or by increasing favourable ones. Consequently, variant peptides have a significantly better capacity to boost weak immune responses than their naturally occurring counterparts. Amino acid substitutions within defined HIV epitopes, such as those within genes encoding structural (*gag*) or enzymatic (*pol*) proteins, stimulate stronger immune responses [18]. With a lower mutation rate than the *env* gene, choosing epitopes from *gag*- or *pol*–derived HLA-restricted immunodominant proteins would provide optimal and prolonged CD8^+^ T cell responses [26]. Though poorly immunogenic on their own, delivering heteroclitic peptides in combination with a monophosphoryl lipid A (MPA) and alum formulation or oil-based adjuvant will stimulate the host immune system and trigger a response favouring cytotoxicity [27]. Incorporating heteroclitic peptides in HIV therapeutic vaccination strategies has the potential, in ways that native peptide vaccines lack, to invigorate HIV-specific CD8^+^ T cell responses, thwart T cell exhaustion and reduce viremia in cases of disease progression.

Current HIV management involves combination antiretroviral therapy (cART) targeting various stages of the viral life cycle. Efficacy requires strict adherence and this, coupled with high cost, limits the global use of cART. Although cART has undergone great improvements in reducing toxicity and resistance, stimulating more effective CD8^+^ T cell immunity using unnatural variant peptides offers an attractive alternative or adjunct therapy to cART. If effective, this could lower costs, simplify treatment and reduce the life-long toxicity of daily drug regimens. As there is considerable diversity in how CD8^+^ T cells of different individuals respond to the same variant, large libraries of potential heteroclitic peptides would need to be available for personalized identification of optimum peptides for therapeutic vaccination. Ideally, a higher proportion of CD8^+^ T cells with enhanced cytokine production profiles and superior cytotoxic function would be activated. Additionally, these cells would proliferate and differentiate into more durable effectors than those maintained through responses against reference peptides.

Beyond contributing towards long term viral control, heteroclitic peptides could also play an important role in cure strategies for HIV infection. With the exception of extremely rare cases, HIV has been impossible to cure and recurrence of viremia in patients off cART is attributed to HIV reactivation from viral reservoirs [28–30]. To overcome HIV persistence, a ‘shock and kill’ strategy is proposed, whereby HIV is driven out of latency (‘shock’), infected cells are subsequently targeted by HIV-specific immunity and nascent replication is halted by cART (‘kill’) [31]. However, the efficacy of the T cell response required for this strategy is generally insufficient in patients with chronic infection, especially those experiencing T cell dysfunction and exhaustion. Thus, combining a therapeutic strategy that invigorates HIV-specific CTL with the shock and kill approach will be required to efficiently eliminate cells shocked into viral replication, limit do novo viral spread and enable HIV eradication.
